# Hysteria in Early Life*Abstract of paper read before the Philadelphia County Medical Society, June 23, 1897.

**Published:** 1897-11

**Authors:** Augustus A. Eshner

**Affiliations:** Philadelphia, Pa., Professor of Clinical Medicine in the Philadelphia Polyclinic; Physician to the Philadelphia Hospital, etc.


### HYSTERIA IN EARLY LIFE.*

By AUGUSTUS A. ESHNER, M.D., PHILADELPHIA,

Professor of Clinical Medicine in the Philadelphia Polyclinic; Physician to the Philadelphia Hospital, etc.

Through the labors of the French school of neurologists in particular, hysteria has been given the recognition it deserves as a distinct, fixed clinical entity, with a train of symptoms as characteristic as those of any of the acute specific infections or intoxications. That the disorder has a pathology of its own I have no doubt the results of future investigation will demonstrate, but as yet we need more knowledge, especially in the domain of physiologic and pathologic chemistry, before we may hope for a solution

*Abstract of paper read before the Philadelphia County Medical Society, June 23, 1897.

of this aspect of the problem. From both inference and analogy it seems not unreasonable to believe that hysteria depends essentially upon metabolic or nutritional changes in the cellular elements of the central nervous system, in consequence of which there may result alterations in function and changes in relation, whence arise the varied and protean symptoms of the developed disease. It may not be extravagant to hope that further refinement in staining methods, in which there has been so remarkable an advance in the past decade, may make possible the detection of changes in nerve-cells that at present elude closest scrutiny by existing means of investigation.

Though we retain the name, which perpetuates the original erroneous conception of its pathology, we have learned that hysteria may exist not only in women deprived of their uteri, but even in men as well. Hysteria respects neither sex nor age, although by far more common in females than in males, and comparatively rare in early and in late life. The explanation of these differences must be looked for in the varying susceptibility and receptivity of the nervous system with regard to those influences to which, in a general way, we have learned to attribute etiologic activity. According to statistics cited by Lloyd in "A Text Book of Nervous Diseases" (edited by Dercum), hysteria is most common in women at the age of twenty years. Briquet found one-fifth of the cases in the female sex to occur before puberty; and rather more than one-third between the ages of fifteen and twenty. Batault found the disease most frequent in men between the ages of ten and twenty. Mills, in the "Cyclopædia of Diseases of Children (edited by Keating), reports a case of catalepsy or automatism, in a girl two years old, and refers to a similar case, reported by Jacobi, in a child three years old. He cites also a case of hysterical paralysis in a girl eighteen months old, reported by Gillette. The evidence goes to show that hysteria, while perhaps not so uncommon in childhood as it appears to be, is yet sufficiently so to warrant the report of a small group of cases illustrating some of the phases of the disease as it appears in early life, as well as some of the difficulties and doubts that at times attend its recognition. The causes, symptoms, course and treatment of hysteria are much the same in children as in adults, except in so far as these are influenced by modifications dependent upon differences in mental and physical growth and development.

*Case 1.*—N. B., a girl, nine years old, came to the clinical service of Dr. Lewis, at the Orthopædic Hospital and Infirmary for Nervous Diseases, on April 14, 1897, with a history of several times daily, for two years, going through a series of peculiar movements, which consisted essentially in dropping the arms and spreading out the hands as if to catch herself in the act of falling, without loss of consciousness. She had had, besides, three convulsive seizures, in which she kicked vigorously, rolled up her eyes, etc., and in which it was thought that consciousness was lost. In some of the attacks urine was passed involuntarily, but the anal sphincter was continent and competent. In none was the tongue bitten. The child was exceedingly emotional, crying readily, and being subject to attacks of causeless laughter. At times there was headache. The knee-jerks were preserved and station was steady. The dynamometric record was ten in each hand. There was no sensory derangement. The family history showed no evidence of neurotic predisposition. The little patient herself had never been seriously ill.

Under date of June 21, 1897, it is noted that the onset of the attacks followed the eating of a quart of peanuts. At that time the child had a continuous series of convulsions for two days. At present it is said that the seizures are repeated at intervals of five minutes during the day, although but one occurred during the quarter of an hour that the child was under observation, and in this, which lasted but a few seconds, the child doubled up on its mother's lap slightly and dropped its head forward. It might have fallen had it not been supported. The attacks take place not only in the presence of others, but also when the child is alone, and she has injured herself in several. They are superinduced by the ingestion of such articles of food as meat, cabbage, tea, coffee, etc.

The child is exceedingly pallid; its digestion is poor and its bowels constipated. Pin-prick is everywhere readily appreciated. The heart is said to beat rapidly at the close of the attacks, but auscultation fails to disclose evidence of organic disease. Dr. A. G.

Thomson was unable to detect any abnormality of fundus or of muscular balance. Hypnosis was attempted, but apparently without success.

While some of the features of this case are strongly suggestive of hysteria, others are not less strongly suggestive of epilepsy, and without further study the discrimination is by no means easy. I admit that the criticism may be justly made that the doubt surrounding the diagnosis should be sufficient to exclude the case from this report, but if there is no other justification for its inclusion, I may be permitted to retain it in order to emphasize the difficulty with which the differentiation of the two diseases is sometimes attended, and to dwell briefly upon the fact that the same patient may be the victim of both.

This difficulty in diagnosis, and especially as it occurs in children, is illustrated by a case that came under observation only to-day in the clinical service of Dr. Lewis, at the Orthopædic Hospital.

*Case 2.*—A nervous mother brought her little daughter of seven to learn what was the matter with her. The child was pale and illy nourished, but had no definite complaint. She had fallen about a month ago, striking her nose and suffering a copious epistaxis, which had been repeated some three weeks later. About two weeks ago the child was seized with severe headache, followed by high fever and delirium, continuing for two or three days. After the lapse of a week, the symptoms reappeared, lasting now, however, only throughout the night. Inquiry elicited the fact that two year ago the child had suffered from "congestion of the liver," in the sequence of which she was unable to walk for a period of some weeks. Gradually, with careful training, the power of locomotion returned. There had never been a convulsion or loss of consciousness, and there was no undue laughter or weeping.

Needle-prick on the right hand was less readily appreciated than upon the left; and the point of the needle was said to be felt upon the right side of the face as a piece of hot iron. Some doubt may be felt as to the results of this sensory examination, inasmuch as the responses of the child appeared to be influenced by the tendency of the questions. However this may be, the girl submitted bravely to painful impressions capable of causing an older person to wince.

The knee-jerks were preserved; the gait was normal; the heart was rather overacting, though entirely rhythmic, and its sounds were clear. Hypnotism was attempted, and appeared successful. At one time in the course of this procedure the child, when directed by her mother to open her eyes, maintained that she was unable to do so. I have ventured to stamp this case with a diagnosis of hysteria, though fully alive as to its vulnerability and the possibility of deception on the part of both patient and clinician.

*Case 3.*—S. H., eleven years old, was brought to the clinical service of Dr. Lewis Brinton, at Howard Hospital, by her mother, a music teacher, having two other children, and herself profoundly neurasthenic, with the statement that the child was suffering from spinal trouble, which manifested itself by the appearance of a swelling at the back of the neck. The mother related that she had suffered similarly during her pregnancy with this child, and this condition in her was attributed to "falling of the womb." Both mother and child suffered from time to time from sick headache, with nausea and vomiting. The general condition of the little patient was stated to be sometimes better and sometimes worse. She was said to be quick-tempered and self-willed, and she cried and screamed at times, particularly if not permitted to have her own way; although there had never been a convulsion or loss of consciousness. At times also there was undue laughter. For various reasons, but especially on account of her emotional mobility, the child had not attended school regularly. Her appetite was described as ravenous; the bowels were regular and digestion was good. When headache and nervousness were especially marked the child vomited repeatedly, but improvement ensued in the course of some hours, following the taking of food. Sleep was variable, although as a rule it was good. The knee-jerks were preserved and common, and painful sensibility appeared to be intact. The pupils were full, equal, regular and reactive to light. The action of the heart was rhythmic, its sounds clear. Hypnosis was attempted on two occasions, but without success.

*Case 4.*—L. S., a colored girl, fourteen years old, complained of pain and soreness in the region of stomach, which proved to be sensitive to touch. She was unable to skip and jump and play like other children on account of the resulting distress. She suffered from nausea occasionally, unattended, however, with vomiting. There was complaint of a good deal of headache, although correcting glasses were worn. There were also present tick-like movements of the eyelids. The appetite was good, the tongue coated, the bowels constipated. Menstruation had appeared for the first time some nine months previously, and although a little irregular was unattended with pain. During the preceding month there had been suppression of urine for three or four days on two or three occasions. The urine itself was said to be clear and yellow. For two years the girl was subject to what were described as faints, in which she did not fall, though she seemed to lose consciousness. In these she clenched her fists, and on one or two occasions she kicked, but she had no well-defined convulsion. The duration of the attacks was said to range from five to thirty minutes. At the conclusion of the attack the child seemed exhausted and she felt drowsy. There was at no time undue laughter or causeless weeping. The attacks recurred two or three times a week, at intervals of two or three weeks. There had been a free interval of as long as three months. No definite cause could be assigned for the attacks, though they were associated in the mind of the mother with the function of menstruation, to which, however, their frequency bore no apparent relation. Nothing also was known that seemed to be capable of inhibiting or absorbing the attacks. The child did fairly well at school, although apparently without ambition. There was no history of similar disease or of other form of nervous disorder in the family. The child had never suffered from any serious illness. She was considered sensitive. The knee-jerks were preserved, the patient jumping when the patellar tendon was struck and also in anticipation of a blow that was threatened but not struck. There was no gross sensory derangement. The heart displayed no abnormality. Dr. C. Y. White, by whom the patient was referred to Dr. Brinton's clinic at Howard Hospital, informed me that the patient was susceptible to hypnotism, but her failure to return prevented further study of the case.

Although I believe this case to be one of hysteria, it would appear to be the part of wisdom to withhold for the present a final diagnosis, in order that further study and perhaps personal observation of one of the attacks may yield evidence of such a character as will permit of an unequivocal and unreserved conclusion.

*Case 5.*—R. F., a schoolgirl, fourteen years old, applied at the Orthopædic Hospital and Infirmary for Nervous Diseases, in the clinical service of Dr. S. Weir Mitchell, on July 3, 1896, with a history, having been frightened some eight months before by masqueraders. She fell to the ground and was unconscious for three hours. Her jaws were locked and she breathed heavily. After consciousness returned she went to sleep and remained in bed for several days on account of weakness. Some two months later, following a recitation, the girl lay down and became unconscious and exhibited occasional twitches of the muscles. This attack lasted for three or four hours, and again several days were spent in bed. A third attack occurred after an interval of three months, without known cause. In this also the girl lay down, appeared unconscious, with her eyes open, and became rigid, but she did not bite her tongue. This attack continued for four or five days, during which the patient regained consciousness at times for periods of fifteen minutes. At other times she appeared as if dead, being unable to see or talk. She took a little food, both liquid and solid. At the time of application the attacks recurred almost weekly, lasting for three or four days at a time. The seizures were characterized by rigidity rather than active movement. After some of the earlier attacks there was inability to see, walk or talk. In some of the attacks the patient scratched herself, in some she pulled her hair, in some she kicked at those about her, and in some she made attempts to bite. In some she groaned a good deal, and in others the rectal and vesical contents were passed incontinently. Some of the attacks had occurred while the patient was alone aud some at night in bed. Between the attacks she was often dull, despondent and uneasy. At times, and especially following the attacks, she cried freely and at times she laughed unduly. At times, further, she was unusually obstinate. Appetite, digestion and sleep were good, and the bowels were regular. The knee-jerks were preserved and the pupils were full, equal, regular and reactive to light. There appeared to be general diminution of sensibility to pin-prick. Of the family history it need only be said that the mother, fifty-three years old, was a nervous invalid. The patient herself had had measles and whooping-cough in childhood, and from time to time suffered from bilious attacks. Menstruation appeared first at the age of thirteen and was irregular and painful. In the intermenstrual periods pain and swelling over the left ovarian region were complained of. Prior to the present illness there had never been a convulsion.

The hysterical nature of this case appears so obvious that further comment seems uncalled for.

*Case 6.*—M. M., a schoolgirl, fourteen years old, applied at the Orthopædic Hospital and Infirmary for Nervous Diseases, on September 14, 1896, in the clinical service of Dr. Wharton Sinkler, on account of a coarse, rapid tremor of the right upper extremity, most pronounced in the hand, which had made its first appearance without known cause, a month before, ceasing after a week and being resumed after an interval of three weeks. The movement was least marked during rest and appeared to be increased on voluntary movement. It ceased during sleep. No derangement of sensibility and no limitation of the visual fields could be made out on superficial testing. The dynamometric record was 6 on the right and 23 on the left. The knee-jerks were feeble. The mother of the child was distinctly neurotic. The little patient herself had had four attacks of chorea, two involving the right side and two the left, the first at the age of six years and the last at the age of nine.

Under date of June 21, 1897, it is noted that the tremor has disappeared and reappeared thrice since the previous record. Both the girl and her mother consider her well at present, although on investigation it is found that the right hand is tremulous when extended, and inquiry reveals the fact that the shaking appears on excitement or after muscular activity, but only, or at least only in marked degree, in the right hand. There was no impairment of painful sensibility.

This case illustrates one of the forms of motor disturbance that may attend hysteria. In addition to simple tremor there may be choreoid, or tick-like movements, or spasm or convulsion, or paralysis or paresis.

I have in this communication endeavored by the report of cases to supplement what others have already done in directing general professional attention to the liability of children to suffer from hysteria, and I would further emphasize the importance of its early recognition and intelligent treatment.

---

### HEMATURIA: ITS RELATION TO MALARIAL INFECTION AND THE INGESTION OF QUININE.

In a paper on the above subject, read by Dr. Bransford Lewis, of S. Louis, before the St. Louis Medical Society, strong testimony relating to the injurious, or even fatal effect of quinine administered in cases of malarial hematuria was given.

A number of Southern writers were quoted to the effect that they had never seen a case of malarial hematuria recover in which quinine had been given, and that they had never seen one die in which quinine had not been given.

By personal inquiry and through records of cases reported in the journals during the last ten years, Dr. Lewis had been able to collect the records of four hundred and nineteen cases of which two hundred and seventeen had been over treated with quinine, and two hundred and two without it. Of the two hundred and seventeen treated with quinine, forty-nine died, and one hundred and sixty-eight recovered, giving a mortality of twenty-two and five-tenths per cent. Of the two hundred and two cases treated without quinine, twenty-two died and one hundred and eight recovered, making a mortality of only ten per cent.—less than half as great as that of the quinine cases. But even these figures, the author thinks, do not represent the true difference of mortality-rate between them, which is probably still more favorable for the non-quinine cases; because the non-quinine cases are based upon records of treatment both with and without the drug, whereas the quinine cases are from writers who had only used the one (quinine) form of treatment, reporting favorable terminations, but failing, no doubt, to report many fatal ones that had occurred in their practices.

Non-quinine treatment that had given the best results embraced the objects of elimination, support, and hemostasis without special regard to an anti-periodic effect.

